# Case Report: First report of *Elizabethkingia miricola *infection in a patient with cystic fibrosis

**DOI:** 10.12688/f1000research.14441.2

**Published:** 2018-05-08

**Authors:** Freddy Frost, Dilip Nazareth

**Affiliations:** 1 Liverpool Heart & Chest Hospital, Liverpool, L14 3PE, UK

**Keywords:** Cystic fibrosis, shortness of breath, chest infection, exacerbation, fluoroquinolone

## Abstract

*Elizabethkingia miricola* is a rare non-fermenting Gram-negative rod that has previously been reported to be associated with blood stream and pulmonary abscess infections, but never before in cystic fibrosis (CF). Here we present the first reported case of *Elizabethkingia miricola *infection in a patient with CF and discuss the management options. We describe a patient with CF in whom we observed clinical and spirometric evidence of pulmonary exacerbation with the associated growth of *E. miricola *in sputum culture. The period of clinical instability was observed to coincide with the obtainment of four sputum samples from which *E. miricola *was cultured; improvement was seen following treatment with ciprofloxacin and the subsequent eradication of *E. miricola*. We conclude that *E. miricola* is able to survive in the CF lung and in this case was associated with pulmonary exacerbation. Empirical treatment with fluoroquinolones is appropriate, based on our experience.

## Introduction

*Elizabethkingia miricola*, a non-fermenting Gram-negative rod (NFGNB) was first identified following isolation from condensation water in the Russian space laboratory Mir ^1^. Originally identified as belonging to the *Chryseobacterium* genus, it has since been re-classified and is closely related to *Elizabethkingia meningoseptica* (previously *C. meningosepticum*). *E. miricola* has been demonstrated to be pathogenic, with reports of bacteraemia resulting in sepsis and pulmonary abscesses ^2, 3^. Here, we report the presence of *E. miricola* in the sputum of a patient with cystic fibrosis (CF). To our knowledge, this is the first reported case of *E. miricola* infection in CF. Herein, we discuss the case itself and the literature surrounding this bacterium to help guide clinicians faced with similar clinical scenarios.

## Case report

A 49-year-old male with a diagnosis of CF presented to his routine CF outpatient department complaining of feeling generally unwell. He reported increased cough, but this was predominantly non-productive. There was a drop in lung function, from a baseline forced expiratory volume in one second (FEV1) of 2.39 l (65% of the predicted volume) to 2.19 l (60% predicted). A sputum sample was obtained following chest physiotherapy and sent for routine culture on blood agar, chocolate agar, Sabouraud agar, Staphylococcus agar, m-Kleb agar and cepacia selective agar. Given the non-specific symptoms and mild drop in FEV1, it was agreed that no immediate treatment was required and a follow-up in 4 weeks’ time was arranged.

Co-morbidities of the patient included osteoporosis and pancreatic insufficiency; he was also receiving maintenance treatment for allergic bronchopulmonary aspergillosis (ABPA) in the form of oral anti-fungal therapy and long-term low-dose oral corticosteroids. Cultured respiratory samples in the previous year had consistently grown non-epidemic *Pseudomonas aeruginosa*. The patient was receiving a continuous alternating inhaled anti-pseudomonal antibiotic regime in the form of tobramycin (TOBI 300mg BD) and aztreonam lysine (Cayston 75mg tds). The diagnosis of CF was made in adulthood and was based upon the presence of bilateral upper zone bronchiectasis on a chest CT scan and a raised sweat chloride level following a sweat test. Initial genetic testing revealed one copy of the F508del mutation, a second mutation was not identified despite extended screening. Family history included a younger sister who had died aged 23 years from pancreatitis. Serum immunoglobulin testing at the annual screen performed two months prior was within normal limits aside from a chronically raised IgG anti-aspergillus of 154 mg/L.

**Figure 1.**
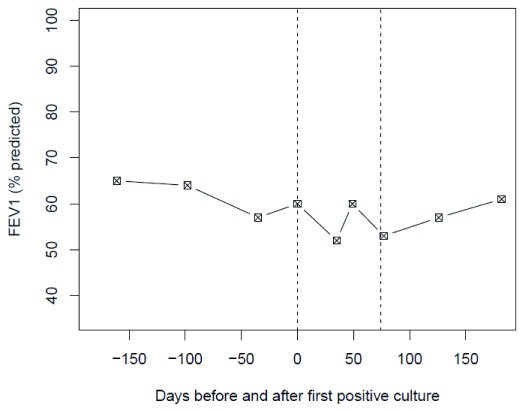
Lung function before and after positive sputum culture for *Elizabethkingia miricola.* Dotted lines represent the time period between which four sputum cultures were positive for *E. miricola*.

A sputum sample taken at the clinic appointment was positive for *P. aeruginosa*, and extended 10 day incubation on cepacia selective agar resulted in isolation of a cream coloured colony. The colony was identified as *Elizabethkingia miricola* by MALDI-TOF (matrix-assisted laser desorption/ionisation time-of-flight) mass spectrometry. At the next appointment, worsening symptoms were observed, including increasing shortness of breath, wheeze and productive cough. There was a further drop in FEV1 to 1.91 L (52% predicted) ( Figure 1). An oral course of chloramphenicol (500 mg four times a day) along with prednisolone (30 mg daily) for 2 weeks was commenced and a further sputum sample was obtained. Chloramphenicol was chosen empirically based on a previously observed clinical response to the agent and also patient preference to avoid ciprofloxacin due to skin photosensitivity. The sputum culture taken prior to treatment initiation was again positive for *P. aeruginosa* and *E. miricola*.

A further 4 weeks later, symptoms were somewhat improved and FEV1 had increased to 2.19 l (60% predicted). However, another 2 weeks later, symptoms deteriorated again, with an associated decline in lung function (FEV1, 1.95 l; 53% predicted). Sputum cultures from the previous encounter were again positive for *P. aeruginosa* and *E. miricola*. Sensitivities from previous samples revealed *E. miricola* resistant to meropenem and ceftazidime, but sensitive to piperacillin/tazobactam and ciprofloxacin (CIP). A 2-week course of oral CIP (750 mg thrice daily) was therefore commenced.

The patient noted an improvement in symptoms and at the next clinic appointment FEV1 had improved to 2.08 l (57% predicted). Sputum then grew *P. aeruginosa* and yeast only. A further four subsequent sputum samples 1, 4, 8 and 12 months later have grown *P. aeruginosa* but no *E. miricola*, and lung function returned towards baseline.

## Discussion

Here, we describe an adult with CF in whom we observed clinical and spirometric evidence of pulmonary exacerbation, with associated growth of *E. miricola* in sputum culture. The period of clinical instability was observed to coincide with four sputum samples culturing *E. miricola* and improvement was seen with treatment. This is the first report of *E. miricola* in an individual with CF, meaning this report should therefore be relevant to all CF clinicians and microbiologists involved in the care of people with CF.

*E. miricola* has been described in a number of healthcare settings, but not previously in CF. One of the first reports of *E. miricola* infection was of ** positive growth in blood and sputum cultures of a septic patient whom had recently undergone a stem-cell transplant for mantle-cell lymphoma. Since then it has been reported in only a handful of cases, including septicaemia in a young patient with alcoholic pancreatitis and in the sputum of a septic patient with pulmonary abscesses ^2, 3^. A degree of immunocompromise is a unifying feature in these cases, and the long-term oral corticosteroids required for treatment of ABPA in the present case may have predisposed to infection with *E. miricola*. However, more recently, *E. miricola* has also been identified as causing a UTI in an immunocompetent adult ^4^.

All case reports of *E. miricola* infection mentioned above report identification by MALDI-TOF ^2– 4^. MALDI-TOF has been widely adopted for bacterial identification, facilitating diagnosis quickly and reliably. In the CF setting, MALDI-TOF has also been shown to be particularly useful in identifying non-fermenting gram-negative bacteria, for which classification can be difficult using conventional phenotypic approaches ^5^. Given the increasing use of MALDI-TOF in clinical microbiology laboratories, identification of NFGNB infections is likely to rise. Hence, establishing the optimal initial management strategies for these infections is important.

In this case, initial empirical treatment with oral chloramphenicol did not clear the infection, but treatment with oral CIP (based on culture sensitivities) successfully treated the exacerbation. Eradication of *E. miricola* was also observed, with contemporaneous clinical improvement. Notably, the sputum culture sensitivities revealed that *P. aeruginosa* was resistant to CIP, further supporting the idea that *E. miricola* had a pathogenic role. Our experience of treatment with a fluoroquinolone is in keeping with that of previous reports, in which *E. miricola* bacteraemia has been associated with sensitivity to levofloxacin and/or CIP, both of which resulted in successful treatment ^2, 6, 7^. Susceptibility to co-trimoxazole (SXT) has also been reported, and it would seem that treatment with fluoroquinolones or SXT is an appropriate empirical strategy.

## Conclusion

*E. miricola* appears to have the potential to grow in the CF lung and can be associated with pulmonary exacerbation. Given the paucity of information on *E. miricola* infection in CF, we hope that the case report and literature review herein are relevant to CF clinicians and microbiologists alike. Treatment based on culture sensitivity is recommended, but empirical treatment with fluoroquinolones may be an appropriate initial strategy if there is suspicion of pathogenicity.

## Consent

Written informed consent for the creation and publication of this report was obtained from the patient.

## Data availability

The data referenced by this article are under copyright with the following copyright statement: Copyright: © 2018 Frost F and Nazareth D

Data associated with the article are available under the terms of the Creative Commons Zero "No rights reserved" data waiver (CC0 1.0 Public domain dedication). 

All data underlying the results are available as part of the article and no additional source data are required.
