# Supplementary material for: Case Report: First report of Elizabethkingia miricola infection in a patient with cystic fibrosis
Source: F1000Res. 2018 May 8;7:440. Originally published 2018 Apr 10. [Version 2] doi: 10.12688/f1000research.14441.2 (PMC6058470; doi:10.12688/f1000research.14441.2)
Supplement: Peer review history

# Peer review: Referee response for version 2

**DOI:** 10.5256/f1000research.16111.r36139

**Published:** 2018-07-20

**Authors:** Jamie Duckers

**Affiliations:** 1 Cardiff and Vale University Health Board, Cardiff, UK

This is an interesting and well written case describing isolation of *Elizabethkingia mircola* in a patient with Cystic Fibrosis during a period of clinical deterioration.

 The authors have addressed previous referees comments. I would add that there is a paper by Dervla et al. 2018 in Diagnostic Microbiology and Infectious disease which highlights the prevalence of an *E. miricola* cluster over other *Elizabethkingia* species among UK cystic fibrosis patients. However the paper by Dervla et al. concentrates on identification rather than clinical course and management which Frost et al. describe.

 I would point out that in vitro sensitivity of *pseudomonas* will not always predict in vivo response and the ciprofloxacin used could have actually treated the *pseudomonas* as well as the *E. miricola* thus making it more difficult to be definitive that the clinical decline was entirely due to *E. miricola* and not the *pseudomonas*.

I have read this submission. I believe that I have an appropriate level of expertise to confirm that it is of an acceptable scientific standard.

# Peer review: Referee response for version 2

**DOI:** 10.5256/f1000research.16111.r33793

**Published:** 2018-05-08

**Authors:** Andrew M. Jones

**Affiliations:** 1 Manchester Adult Cystic Fibrosis Centre, University Hospital of South Manchester, Manchester, UK

The authors have revised the article and addressed all the comments satisfactorily.

I have read this submission. I believe that I have an appropriate level of expertise to confirm that it is of an acceptable scientific standard.

# Peer review: Referee response for version 1

**DOI:** 10.5256/f1000research.15719.r33065

**Published:** 2018-04-30

**Authors:** Andrew M. Jones

**Affiliations:** 1 Manchester Adult Cystic Fibrosis Centre, University Hospital of South Manchester, Manchester, UK

This is the first case described as far as I am aware of Elizabethkingia mircola in a patient with cystic fibrosis.The authors describe isolation of Elizabethkingia mircola in association with clinical deterioration of a patient, and subsequent clinical improvement and eradication of the organism following quinolone therapy.

 The report could be further improved by addressing the points below:

- The authors state in the first paragraph that the patient had a cough that was unproductive, but in contrast also state that a sputum sample was obtained.
- The report states that the patient was receiving treatment for ABPA; it is important to know what exactly the treatment was and in particular if this could have caused immunosuppression and lead to increased susceptibility to unusual infections.
- The authors state the patient was heterozygous for F508del; did they identify the other CF gene?
- The authors state Elizabethkingia mircola was isolated after extended incubation: what culture media was used and how long was the extended incubation before Elizabethkingia mircola was isolated?

I have read this submission. I believe that I have an appropriate level of expertise to confirm that it is of an acceptable scientific standard, however I have significant reservations, as outlined above.

# Response

**Published:** 2018-05-01

**Authors:** Freddy Frost

**Affiliations:** Liverpool Heart & Chest Hospital NHS Foundation Trust, UK

We are grateful to Professor Jones for his time and review of our case-report. We have moved to clarify the points he has raised. In particular we have reviewed the electronic patient record and clarified that sputum sample was actually obtained by our physiotherapist. We have also expanded on the treatment for ABPA which did include low-dose oral corticosteroids and have discussed the relevance of this in our discussion. 

We have confirmed that only one gene was present despite extended genotype screening, but highlighted the reasons for making a robust clinical diagnosis of CF. We have also confirmed that growth was seen on the cepacia selective plate.

# Peer review: Referee response for version 1

**DOI:** 10.5256/f1000research.15719.r33066

**Published:** 2018-04-23

**Authors:** Simon C. Langton Hewer

**Affiliations:** 1 Paediatric Respiratory Medicine, Bristol Royal Hospital for Children, Bristol, UK

I agree that this appears to be the first report of this organism in CF and as such this is a relevant and important article and so should be published. The Case Report para 2 states the patient was heterozygous for CFTR mutation F508del but does not give the other mutation. Readers (including me) would be interested to know the second mutation so this should be stated.

 Para 4 states treatment of P aeruginosa and E miricola took place with oral chloramphenicol. This is an unusual choice as P. aeruginosa would usually be treated with ciprofloxacin or IV therapies and/or nebuliser amino glycoside. It would be helpful to know why chloramphenicol was chosen and whether any neb antibiotics were used as well (which is likely the case). 

 These details should be included to give a more complete picture of the case.

I have read this submission. I believe that I have an appropriate level of expertise to confirm that it is of an acceptable scientific standard, however I have significant reservations, as outlined above.

# Response

**Published:** 2018-04-24

**Authors:** Freddy Frost

**Affiliations:** Liverpool Heart & Chest Hospital NHS Foundation Trust, UK

We are very grateful to Dr Langton-Hewer for his review and comments. In response to Dr Langton-Hewer’s comments:

- Only one gene was identified despite extended testing. The diagnosis of CF was made based on one gene, raised sweat test, upper lobe bronchiectasis and family history. We have now included more detail with regard to this point.
- Oral chloramphenicol was chosen empirically based on previous response and the patients desire to avoid photosensitivity associated with ciprofloxacin. We have no included more detail in the manuscript.
- Nebulised therapy included Cayston and TOBI, these were continued throughout and we have now included this detail within the manuscript
